# Fabrication of Customized Nanogel Carriers From a UV-Triggered Dynamic Self-Assembly Strategy

**DOI:** 10.3389/fchem.2019.00769

**Published:** 2019-11-08

**Authors:** Wuren Bao, Jieran Lyu, Chunlin Li, Jifeng Zhang, Tunan Sun, Xing Wang, Jin Zhou, Dawei Li

**Affiliations:** ^1^School of Nursing, Inner Mongolia University for Nationalities, Tongliao, China; ^2^Clinical Medicine Academy of Shandong First Medical University, Tai′an, China; ^3^Beijing National Laboratory for Molecular Sciences, State Key Laboratory of Polymer Physics and Chemistry, Institute of Chemistry, Chinese Academy of Sciences, Beijing, China; ^4^The 8th Medical Center of Chinese PLA General Hospital, Beijing, China; ^5^Department of Orthopedic Surgery, Tongliao City Hospital, Tongliao, China; ^6^University of Chinese Academy of Sciences, Beijing, China

**Keywords:** nanogel, morphology evolution, UV-trigger, redox-responsive, drug carrier

## Abstract

Recent advances in self-assembled nanogel carriers have allowed precise design of hierarchical structures by a low-cost solution-phase approach. Typically, photochemical strategy on the tailor of morphology and dimension has emerged as a powerful tool, because light-trigger has exceptional advantages of an instant “on/off” function and spatiotemporal precision at arbitrary time. Herein, we report a tunable manipulation of sequentially morphological transition via a “living” thiol-disulfide exchange reaction from a UV-tailored hierarchical self-assembly strategy. By varying the irradiation time, the photochemical method can easily fabricate and guide a series of attractively architectural evolution in dilute aqueous solutions, by which the improving hydrophobicity and sensitive redox-responsiveness endowed these disulfide-linked nanoparticles with remarkable capacities of abundant encapsulation, effective separation, and controlled release of hydrophobic cargoes. Notably, once the exchange reaction is suspended at any point of time by removing the UV lamp, these active sites within the nanogel carriers are instantaneous deactivated and the correspondingly structural transformations are also not conducted any more. However, if the stable inert sites are reactivated as needed by turning on the UV light, the interrupting morphology evolution can continue its previous steps, which may provide a simple and novel approach to fabricating the desired self-assemblies in solutions. With regard to this advanced functionality, various nanogel carriers with customizable structures and properties have been yielded and screened for cancer therapy. Thus, this “living” controlled self-assembled method to program morphology evolution *in situ* is a universal strategy that will pave novel pathways for creating sequential shape-shifting and size-growing nanostructures and constructing uniform nanoscopic functional entities for advanced bio-applications.

## Introduction

Hydrogel particles (microgels and nanogels), composed of a series of microsized or nanosized cross-linking networks, own the high water contents, satisfying structural stability and desirable mechanical property, which has great potentials in applications of biosensing and drug delivery (Stefik et al., 2015; Palivan et al., 2016). Although frequently used methodologies have been reported to prepare hydrogel particles by the polymer polymerization, self-assembly, template-assisted nanofabrication, and microfluidic techniques, the precise and successive fabrication of microgels/nanogels is still challenging because of the difficult design and complex process for tailoring the structures and properties.

The solution self-assembly of nanogels offers an efficient and bottom-up approach on creating various well-defined nanoparticles with significant fields of drug delivery, nanoreactors, photonic detectors, etc. (Lee et al., 2016; Yang et al., 2018). Fabrication of shape-shifting and size-growing architectures appears as an important approach on improving the nanoscience and nanotechnology, because a number of functional nanogel carriers are directly determined by their shapes and sizes. Various approaches on generation of uniform morphologies and desire dimensions are always relied on the fine control of special architectures (chemical composition, geometry, hydrophobic/hydrophilic ratio, surface chemistry, and flexibility), external environments (pH, temperature, light, stress, photon, ultrasound, and ionic strength), and selective cosolvent mixtures (Gröschel et al., 2013; Newkome and Moorefield, 2015; Tan et al., 2015; Hunt et al., 2016; Mason et al., 2016; Zhang et al., 2018). Whereas, these methods are still difficult to delicately manipulate the self-assembled system and veritably observe all kinds of solution-assemblies or pre-assemblies because of the complexity in precise control of intrinsic properties (hydrophobic/hydrophilic ratio, architecture, rigidity, size, etc.) for amphiphiles at any moment once trigging the self-assembly system at the beginning, and hence the simple management of *in-situ* morphology evolution with an instant “on/off” function to tailor the solution-assemblies at arbitrary time is of the utmost importance. Therefore, it is urgent and important to develop some manipulative reactions to regulate the self-assembly systems with a vital criteria that the self-assembly behaviors could be controllably activated, terminated, and interrupted at any time, and should also be reinitiated by trigger of the external stimuli whenever needed. Although the pH-triggered “living” morphology evolution and controlled hydrogels are well-researched based on a thiol-disulfide exchange reaction in our previous works (Wu et al., 2010; Zhang et al., 2012; Wang et al., 2013, 2018a), consequences of harsh pH environments on the modulation of thiol-disulfide exchange reaction and the corresponding self-assembled nanogels are typically overlooked for the biomedical applications.

**Scheme 1:**
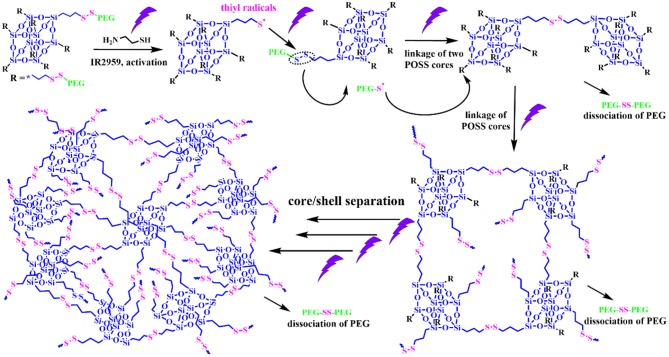
Schematic representation of controlled thiol-disulfide exchange reaction via a UV switcher.

Alternatively, a photochemical reaction is a powerful tool to modulate the thiol-disulfide reaction, which possess unique advantages like remote trigger, variable light intensity, smart on-off function and spatiotemporal precision, realizing the real-time control over the whole photochemical process. Inspired from this intriguing concept, we demonstrate a “living” controlled strategy on successful regulation of the self-assembled evolution *in situ* by manipulating UV switcher to turn on/off thiol-disulfide exchange reaction for an amphiphilic POSS-(SS-PEG)_8_ polymer in dilute aqueous solutions. Scheme 1 illustrated the possible mechanism of UV-triggered thiol-disulfide exchange that can cause the spontaneous dissociation of PEG shells and linkage of POSS cores along with the continuous reaction progress; in this case, the hydrophobic territory was gradually enlarged and topological architecture was continuously evolved. Thus, we found that the rational control of UV trigger and thiol-disulfide reaction time could easily adjust the hydrophobic/hydrophilic ratios and topological structures, thus generating time-dependent morphology transition and size growth during the continuous exchange reaction. Notably, this self-assembled evolution behavior can be interrupted at any time by turning off the UV light and restarted by turning on the irradiation. The rigid POSS backbone and its strong aggregation ability endow the POSS-containing hybrid polymers with hierarchically self-assembly behaviors in selective solvents, presenting the multifarious morphologies and *in situ* assembling process (Zhang and Muller, 2013; Li et al., 2015; Ni et al., 2015; Wang et al., 2015, 2016, 2018b; Dong et al., 2016; Qian et al., 2019). More importantly, unlike previously reported shape-shifting organic nanostructures by tediously adjusting conditions, the morphology conversion in this “living” controlled system, relied on the stimuli-responsive “on/off” reaction, was tunable, continuous, and diversified, by which high-throughput synthesis and fabrication of polymeric colloids are well-realized for biomedical applications. Thus, it is worth carrying out the scientific studies to systemically understand the fundamental principles governing the slow-growth and morphology transition of nanostructures based on such inherent advantages and facile strategies.

## Materials and Methods

### Materials

POSS-(SS-PEG)_8_ (*M*_n,PEG_ = 750 g/mol) was prepared according to the previous work (Wang et al., 2013), DMEM was purchased from Beijing BioDee Biotechnology Co. Ltd. Cell counting kit-8 was purchased from Beyotime Biotechnology. MCF-7 cell lines were purchased from the American type Culture Collection (ATCC, Rockville, MD). Other chemical reagents were commercially purchased and directly used without any further purifications.

### Characterizations

Infrared spectrum was performed on an Excalibur Series FT-IR spectrometer (DIGILAB, Randolph, MA) by drop-casting sample films on the KBr plate. The polymeric compositions were determined by NMR spectra using a Bruker DRX-400 spectrometer with CDCl_3_ as the solvent and tetramethylsilane (TMS) as the internal standard. Transmission electron microscopy (TEM) images were obtained on a JEM-2200FS microscope (JEOL, Japan). A 5 μL droplet of assembled solution was dropped onto a copper grid (300 mesh) coated with a carbon film, followed by drying at room temperature. Scanning electron microscopy (SEM) images were obtained at acceleration voltage of 5 kV on a JSM-6700F microscope (JEOL, Japan). The samples were sputter-coated with a thin layer of Pt for 90 s to make the samples conductive before testing.

### Preparation of POSS-(SS-PEG)_8_@TPE Aggregates

POSS-(SS-PEG)_8_@TPE aggregates were self-assembled as follows: 5 mg of POSS-(SS-PEG)_8_ polymers and 1 mg of TPE-CHO molecules were firstly dissolved in 1 mL of THF, then 4 mL of ultrapure water were added dropwise at the rate of 0.05 mL/min via a syringe pump. After addition of the IR2959 and cysteamine, the exchange reaction and self-assembly behavior were triggered by turning on/off the UV irradiation (365 nm, power density of 3 mW/cm^2^) at a predetermined time.

### Preparation of POSS-(SS-PEG)_8_@DOX Aggregates

POSS-(SS-PEG)_8_@DOX aggregates were self-assembled as follows: 5 mg of POSS-(SS-PEG)_8_, a predetermined amount of DOX·HCl and 1.5 molar equiv of triethylamine were dissolved in 1 mL of DMF at room temperature for 2 h. Then 4 mL of ultrapure water were added dropwise at the rate of 0.05 mL/min via a syringe pump. The stable morphology can be facilely yielded by turning on/off the UV irradiation at a predetermined time, and the various dried DOX-loaded aggregates were finally obtained after dialyzing against deionized water for 24 h (MW cutoff, 4 kDa) to remove the unencapsulated DOX molecules and byproducts.

### CCK-8 Assay

The cytotoxicity of POSS-(SS-PEG)_8_ polymers and DOX-loaded aggregates (after exchange reaction under UV irradiation for 4 and 24 h, respectively) was investigated by CCK-8 assay. Specifically, MCF-7 cells were seeded onto a 96-well plate with a density of 1 × 10^4^ cells per well in 180 μL of DMEM containing 10% fetal bovine serum (FBS) and incubated for 24 h (37°C, 5% CO_2_). The medium was replaced by 90 μL of fresh DMEM medium and then 20 μL of samples with 2–10 mg/mL of the aggregate suspensions in PBS (pH 7.4) were added. After incubation for another 24 h, the culture media were removed from cell culture plates, and 100 μL of fresh culture media and 10 μL of CCK-8 kit solutions were immediately poured into and homogeneously mixed with another 4 h of incubation in a CO_2_ incubator. Finally, 100 μL of self-assembled solutions were put into 96-well plate. The optical density of each well at 450 nm was read using a microplate reader. Cells cultured in DMEM medium containing 10% FBS (without exposure to polymers or aggregates) were used as controls.

## Results and Discussion

**Figure 1:**
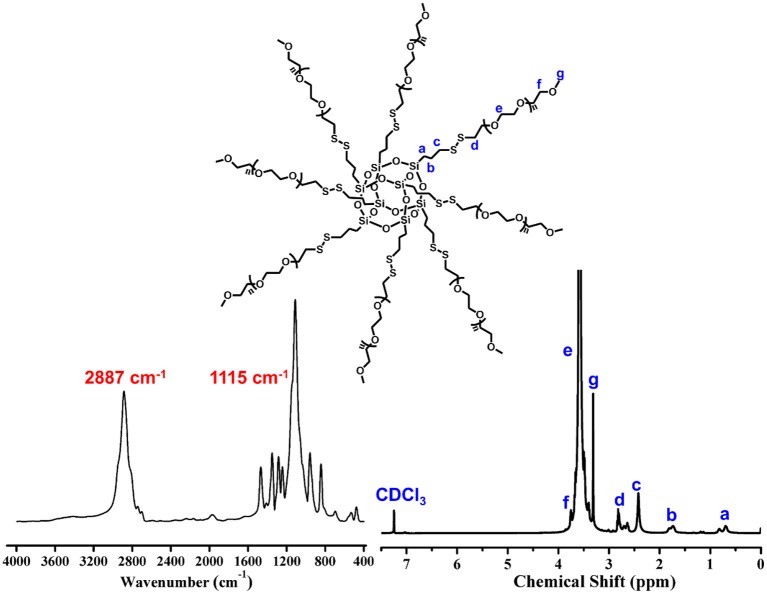
FT-IR and ^1^H NMR spectra of POSS-(SS-PEG)_8_.

The precursor of POSS-(SS-PEG)_8_ was yielded according to our previous work (Wang et al., 2013). IR spectrum give an intuitive observation with the shark peak of 2,887 cm^−1^ attributed to *C-H* stretching of CH_2_ at PEG and strong signal of 1,115 cm^−1^ belonged to the Si-O-Si at POSS. ^1^H NMR spectrum showed a preferable assignment of all characteristic peaks (Figure 1), demonstrating uniform disulfide-crosslinked core/shell structure with a POSS core and eight peripheral PEG shells.

**Figure 2:**
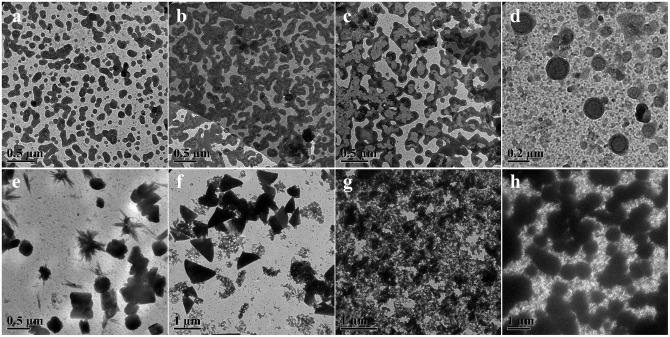
TEM images showing morphology and size evolution with a concentration of 1 mg/mL aqueous media under UV irradiation at **(a)** 0.5, **(b)** 1, **(c)** 4, **(d)** 8, **(e)** 24, **(f)** 32, **(g)** 40, and **(h)** 48 h.

As a representative dynamic bond, disulfide bond could undergo the thiol-disulfide exchange reaction with thiol groups by an anion-mediated or a radical-mediated mechanism under the alkali solutions or irradiation conditions (Xia et al., 2016). Compared to the harsh pH solution to tailor the exchange reactions, UV-triggered radical-mediated behaviors possessed remote manipulation, variable light intensity, smart on-off function and spatiotemporal precision capacities, which could easily achieve the real-time control over the whole photochemical process. Thus, we utilized the IR2959 as a photo-initiator to adjust the radical-centered disulfide exchange reaction by varying the irradiation time followed with concomitant changes in the morphology. On account of high-effective UV-triggered thiol-disulfide exchange reaction, 1 mg/mL concentration of POSS-(SS-PEG)_8_ polymers was applied to reduce the thiol-disulfide exchange reaction without the gelation process in dilute solutions. As shown from Figures 2a,b, the emergence and growth of cylindrical micelles were observed under UV irradiation within 1 h. Shortly, the close-grained networks were generated because the UV-triggered multiple reactive sites were guided by the simultaneous multi-thiol-disulfide exchange reactions in Figure 2c. The existence of intermediates indicated the impossibly homogeneous exchange reaction and ongoing self-organization process (Figure S1a). The increase of fiber clusters in length and width proved the oriented morphology transformation toward the minimization of overall free energy of system during the self-assembly process, in which the strong POSS aggregation capacity to assemble into layered crystals may drive the chain-like structures to evolve into ellipsoids, so the oligomer micellar linkages and junctions could preferentially grow into the long and narrow cylinders at the lowest state of free energy. Then, the thick cylinder-linked networks were slowly dissociated and transferred into vesicles with distinct size gradient distribution (Figure 2d and Figure S1b). After the morphological fusion stage (Figure S1c), the shuttle-shaped sheets were born and grown up, as observed in Figure S1d. Prolonging the irradiation time for cluster fusion, some intermediates and rhombus-shaped nanosheets were inconceivably obtained in Figure 2e and Figure S1e. After UV irradiation for 24 h, the square sheets collapsed and gestated into the triangle-shaped plates in Figure 2f and Figure S1f. The formation of well-defined 2D nanosheets may ascribe to the ordered arrangement of rigid POSS-embedded macromolecules into layers driven by hydrophobic forces. In addition, the fractured segments may also be induced by the degradable nanoparticles under the long periods of irradiation, because the irradiation had more chances to break down the high-density disulfides; in this case, the thicker cluster aggregates could degrade into the shuttle-shaped sheets and other small chips. When the hydrophobic/hydrophilic ratio was substantially increased for a longer time, the morphology evolution come to an end, ultimately forming the silkworm particles with augmented dimension and density (Figures 2g,h, and Figures S1g,h). Overall, the morphology evolution *in situ* from cylindrical micelles to vesicles to clusters to various 2D nanosheets and finally to silkworm particles was well-programmed in sequence with the gradually increased dimensions based on ongoing UV-triggered exchange reaction and ever-changing self-assembled environments.

**Figure 3:**
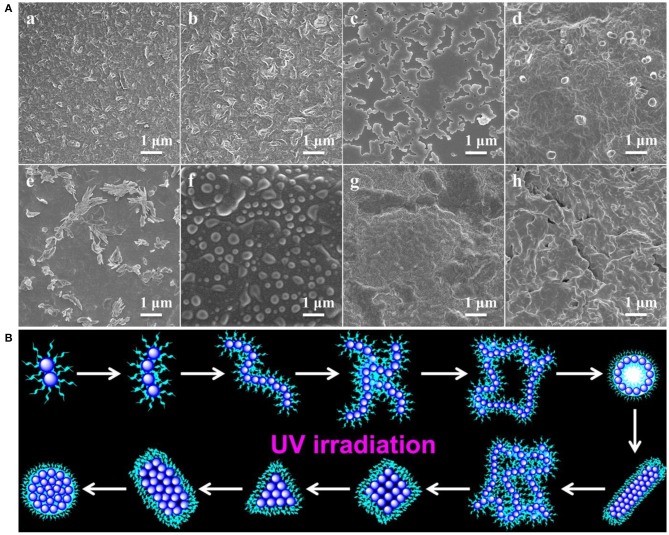
**(A)** SEM images showing morphology and size evolution with a concentration of 1 mg/mL aqueous media under UV irradiation at (a) 1, (b) 2, (c) 4, (d) 8, (e) 12, (f) 24, (g) 36 and (h) 48 h; **(B)** Schematic representation of morphological evolution.

**Figure 4:**
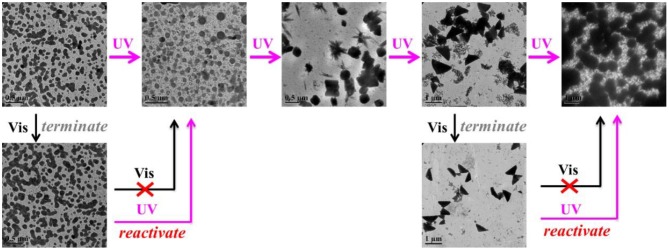
TEM images of *in situ* morphology evolution with UV-responsive “on/off” reaction function.

SEM images in Figure 3A gave additional evidence to testify this UV-controlled morphology evolution. UV-controlled self-assembled transition possessed instant a “on/off” function that can facilely obtain the desired shape and size with high stability in the aqueous solutions only by simple removal of the UV illumination within a predetermined time. Figure 3B vividly depicted the summary representation of morphology and size evolution, intuitively presenting this traceable morphological growth, fusion and transition. It was mentioned that although the size of nanogel is enlarged theoretically, the UV-triggered multiple reactive sites could lead to the simultaneous multi-thiol-disulfide exchange reaction, which may make the random thiol-disulfide exchange, poor-selective core/shell separation and ill-defined size evolution. In addition, UV irradiation could destroy the high-density disulfides incidentally so that the size variation was basically irregular though the morphological transition was well-performed thermodynamically in dilute aqueous solutions. More importantly, once the thiol-disulfide exchange reaction was suspended at any point of time by turning off the UV switcher, the thiyl radicals of self-assemblies were quickly deactivated into the thiols and no further exchange reaction and morphology evolution were conducted any more. However, these stable inert thiols could be reactivated whenever restarted the UV lamp, and the trapped morphology evolution could return back on its right track. For example, when the polymer solution was exposed to the UV irradiation in Figure 4, the short cylindrical micelles were formed around 1 h later. Once removing the irradiation, the inactivation of thiol-disulfide exchange could interrupt the morphology evolution and keep the cylinders for a long time. After turning on the UV irradiation, the reactivation of exchange reaction impelled the morphology transformation to reboot the following process as before. Similarly, stable triangle-shaped plates and other morphologies of nanogels could also be achieved by the suspension of the self-assembled evolution within a predetermined time. Mentioned that the interrupted nanogels could basically maintain their shapes and sizes for more than 3 days in mild environments, which provided a simple and universe approach to yield the conceivable self-assembled nanogels with customized structures and properties through manipulation of the UV switcher within the appointed irradiation time.

**Scheme 2:**
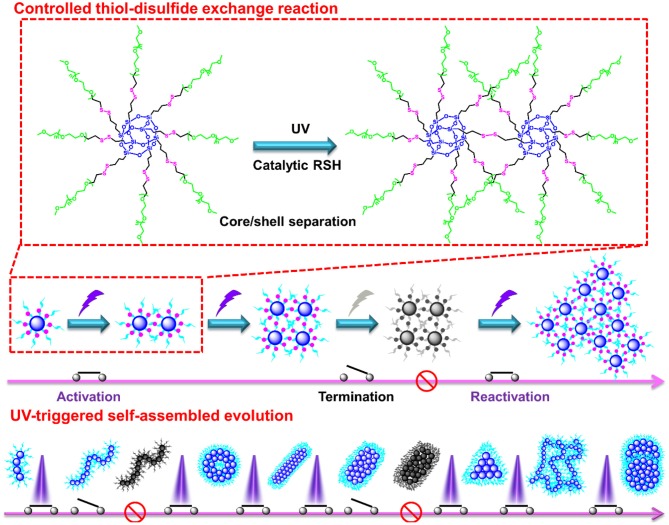
Possible self-assembled mechanism of morphology evolution of nanogels by a UV-switched thiol-disulfide exchange reaction.

The thiol-disulfide exchange mechanism can be utilized to interpret the morphology evolution as a “living” controlled process as depicted in Scheme 2. For the disulfide-linked star-shaped POSS-(SS-PEG)_8_ molecule, UV irradiation could active the thiyl radicals to attack the disulfide linkages within the polymer chains and trigger the exchange reaction in extramolecular disulfides or inner radical combinations, which resulted in the connection of two isolated polymers along with the escape of two well-soluble PEG chains that were regarded as good leaving groups in aqueous solutions. The driving force ascribed to the shifting of the equilibrium toward the stable cross-linked nanogels by the detachment of hydrophilic PEG shells. After a greater degree of thiol-disulfide exchange reactions, more and more crosslinking POSS cores rupturing of PEG segments were induced so as to sustainably decrease the star-shaped polymeric hydrophilicity and cause the regular morphological transition simultaneously. Notice that unlike the flexible hydrophobic polymers to tangle with each other, the POSS-linked cores were rigid and not easy to deformation to decline the free energy of system. So once the re-arrangement of hydrophilic and hydrophobic segments was launched, the repulsive force would compel the multi-architectonical rigid cores to keep spatial distance with adjacent giant POSS molecules, which inevitably enlarged their own hydrophobic spatial areas in spite of no alteration of the hydrophobic components. In other words, although the hydrophilic/hydrophobic ratios had not been changed severely in a period of time, the spatial conformational cores may produce great self-enhancement of hydrophobicity. Therefore, a slight variation of hydrophobic POSS contents and geometries was apt to result in a distinct morphology transition that was consistent with Yan's work (Jin et al., 2012). Besides, the unique POSS aggregation ability brought about the liable axial growth of assemblies, resulting in the emergence and growth of cylinders initially. However, longer UV irradiation may destroy the disulfide linkages so that the cluster nanostructures could dissociate into some kinds of shuttle-shaped sheets or small fragments. Therefore, under the synergistic effects of random thiol-disulfide exchange and photodegradation, many unusual and inconceivable morphology of nanogels came along successively like square, triangle-shaped, rhombus-shaped and silkworm nanoparticles. Based on the above analysis, we concluded that the effects of such unique self-assembly behaviors were mainly depended on below four key factors: ever-changing hydrophobic/hydrophilic ratio, rigid POSS-embedded topology, strong POSS aggregation ability and unpredictable degradation.

**Figure 5:**
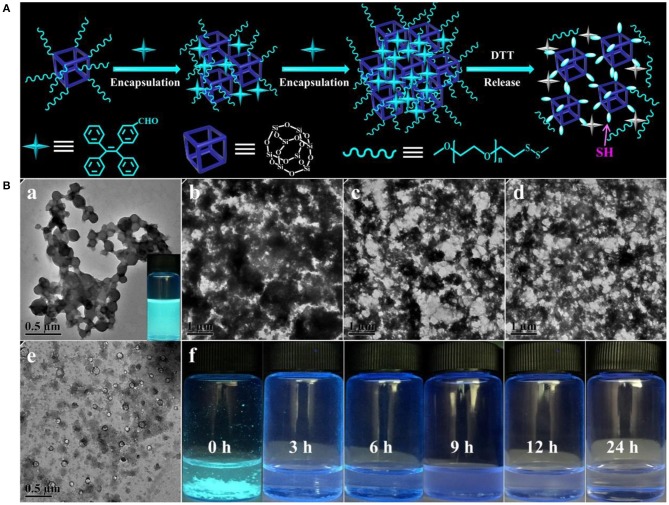
**(A)** Schematic mechanism of the encapsulation, separation and release of TPE-CHO molecules in the process of self-assembled morphology evolution. **(B)** TPE-encapsulated aggregates in the THF/H_2_O mixture containing 10 mM concentration of reductive DTT with the various soaking time.

High loading efficiency and stimuli-responsive property of the nanogel carriers were two pivotal factors for creation of a robust biomedical platform in targeted drug delivery (Guan et al., 2016). On account of the thiol-disulfide exchange reaction that can induce the ever-improving hydrophobicity of the assemblies, TPE-CHO, as a hydrophobic AIE probe, was employed to quantitatively assess the encapsulation and separation efficiency, as schemed in Figure 5A. When the TPE-CHO molecules were added into the THF/H_2_O mixture containing the POSS-(SS-PEG)_8_ molecules, the solution emitted weak fluorescence due to that the TPE-CHO has not efficient capacity to achieve the critical aggregate state of AIE effects. Along with the activation and process of morphology evolution, more and more hydrophobic molecules were gradually encapsulated and aggregated into the self-assembled nanogels due to the strong hydrophobic interactions, leading to the enhancement of fluorescent intensity with obvious AIE attributes (Figure 5B-a). After incubation for more than 24 h, the hydrophobicity of aggregates was dramatically increased and thus the precipitates were slowly formed as observed in Figure 5B-f, which meant the full encapsulation of TPE-CHO molecules into the polymeric nanogels, exhibiting the excellent encapsulation ability for hydrophobic molecules. More importantly, these encapsulated hydrophobic molecules can be separated from the aggregates by resolution of precipitates into good solvents (THF or CHCl_3_) and further recycled by precipitation and centrifugation methods.

The formation of nanogels was attributed to the POSS cores connected by built-up disulfide linkages; therefore, these self-assembled nanoparticles were biodegradable since disulphide bonds could be cleaved by dithiothreitol (DTT). Here, we further evaluated the degradation behavior of the eventually disulphide-linked nanogels in THF/H_2_O mixture containing 10 mM of DTT. On account of the numerous disulfides throughout the backbone of self-assembled nanogels, high concentration of reductive DTT can quickly damage the disulfide bonds to thiol groups and lead to the rapid disruption of aggregates (Figure 5B). As the TPE-loaded aggregates were under high level of reductive solutions, TPE molecules escaped from the disulfide-linked assembled nanogels and the aggregates transferred into the fiber clusters and small vesicles along with the bankrupt of AIE phenomenon, which was intuitively observed from the fluorescent solution (0 h) to turbid solution (3 h) and then to the clear solutions (24 h). Based on the above analysis, we found that although these new molecular constitutes were different from the previous amphiphiles, DTT-induced structural variation can still be understood as a disassembly-induced morphology and size evolution, after all, it triggered the disassembled morphology transition, which would be promising for developing advanced materials for sustained drug delivery system, diagnostic sensor, sensitive detector and opto-electronic transmission.

**Figure 6:**
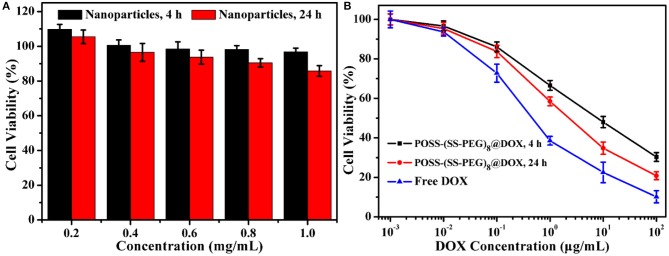
**(A)** Cytotoxicity of two typical kinds of POSS-(SS-PEG)_8_ nanoparticles (after exchange reaction under UV irradiation for 4 and 24 h) to MCF-7 cells following 24 h incubation (*n* = 5 per group). **(B)** Viability assessment of MCF-7 cells with POSS-(SS-PEG)_8_@DOX aggregates (after exchange reaction under UV irradiation for 4 and 24 h) and free DOX as a function of DOX dosages. All the data were presented as the average ± standard deviation.

Similarly, the antitumor DOX drugs were also encapsulated into the nanogels. For example, to overcome the incompatible alkaline environments for living cells, we utilized the *in situ* light-triggered thiol-disulfide exchange reaction with irradiation time of 4 and 24 h. As the exchange reaction induced the continuous improvement of hydrophobic/hydrophilic ratio and variation of architectural topology, the drug loading efficiency could achieve 38.1 and 57.8%. Cytotoxicity of POSS-(SS-PEG)_8_ nanogel carriers (exchange reaction for 4 and 24 h) was investigated by the MCF-7 cells following 24 h of incubation. These two groups exhibited low toxic to MCF-7 cells on account of their biodegradable behaviors with the exposure into high levels of reductive agents. Figure 6A showed that the cell viability was more than 87% even in a concentration of 1.0 mg/mL, presenting the good biocompatibility of nanoparticles. In addition, the viability was also evaluated with two typical POSS-(SS-PEG)_8_@DOX aggregates (exchange reaction for 4 and 24 h) and free DOX as a function of DOX dosage using the MCF-7 cells. Figure 6B displayed that these DOX-loaded nanoparticles possessed high efficiency in cancer cell inhibition. In a low concentration, two DOX-loaded nanoparticles had a similar toxicity as free DOX, but a high concentration of self-assembled nanogel carriers had to be endocytosed to enter the MCF-7 cells, which made free DOX molecules rapider than DOX-loaded aggregates in the whole internalization process.

## Conclusions

In conclusion, we reported a feasible manipulation strategy of self-assembled evolution of nanogels *in situ* using the “living” controlled thiol-disulfide exchange reaction and hierarchically self-assembly behaviors of amphiphilic POSS-(SS-PEG)_8_ polymer. Careful tailoring UV-triggered exchange reaction, the optimal morphology and size evolutions were well-performed, exhibiting the unimolecular, spherical, cylindrical and worm-like micelles, vesicles, rhombus, triangle and square-shaped nanosheets, cluster fibers, hollow spheres and dense nanospheres as well as the gradually dimensional growth process, which ascribed to the sequentially increscent hydrophobic/hydrophilic ratio, variably rigid POSS-embedded topologies and specific POSS aggregation ability. Notably, this morphology and size evolution could be suspended and reactivated as needed at any point of time by simple removal of external UV switcher, and thus the desirable nanogels with various morphologies and sizes were easily gained with good long-term stability in dilute aqueous solutions. The improvement of hydrophobic/hydrophilic ratio endowed these variational assembled nanogels with excellent loading and separation capacities of hydrophobic molecular cargoes. Besides, a large number of disulfide bonds could be degraded under the highly reductive environments, by which the encapsulated molecules were quickly released and the aggregates performed a disassembly-induced morphology evolution. Based on this artificial process technologies, these screened redox-responsive colloidal nanogels have been enabled to fabrication of polymeric nanogel carriers in applications of cancer therapy. This intriguing “living” controlled assembled strategy on fabrication of morphology and size evolution of nanogels is also applicable for other disulfides-linked amphiphilic polymers, which will provide a useful guidance for delicately spatiotemporal control over the artificial superstructures and desired properties of disulfide-crosslinked nanogel carriers. We believe that these simple and smart drug delivery systems could bring about a thorough understanding of complex dynamic process for the amphiphiles nanogels.

## Data Availability Statement

The datasets generated for this study are available on request to the corresponding author.

## Author Contributions

DL, XW, and JZho designed and initiated the research. WB and JL analyzed the data, wrote a draft of the manuscript and completed the biological security of polymeric micelles and DOX-loaded nanoparticles. WB and XW synthesized and characterized amphiphilic polymers. CL, JZha, and TS made important suggestions and helped revising the paper. All authors contributed to discussions on the results and to the finalization of the manuscript.

### Conflict of Interest

The authors declare that the research was conducted in the absence of any commercial or financial relationships that could be construed as a potential conflict of interest.

## References

[B1] Dong X. H. Ni B. Huang M. Hsu C. H. Bai R. Zhang W. B.. (2016). Molecular-curvature-induced spontaneous formation of curved and concentric lamellae through nucleation. Angew. Chem. Int. Ed. 55, 2459–2463. 10.1002/anie.201510524 26773530

[B2] Gröschel A. H. Walther A. Löbling T. I. Schacher F. H. Schmalz H. Müller A. H. (2013). Guided hierarchical co-assembly of soft patchy nanoparticles. Nature 503, 247–251. 10.1038/nature12610 24185010

[B3] Guan M. Ge J. C. Wu J. Zhang G. Chen D. Zhang W.. (2016). Fullerene/photosensitizer nanovesicles as highly efficient and clearable phototheranostics with enhanced tumor accumulation for cancer therapy. Biomaterials 103, 75–85. 10.1016/j.biomaterials.2016.06.023 27376559

[B4] Hunt S. T. Milina M. Alba-Rubio A. C. Hendon C. H. Dumesic J. A. Román-Leshkov Y. (2016). Self-assembly of noble metal monolayers on transition metal carbide nanoparticle catalysts. Science 352, 974–978. 10.1126/science.aad8471 27199426

[B5] Jin H. B. Huang W. Zhu X. Y. Zhou Y. F. Yan D. Y. (2012). Biocompatible or biodegradable hyperbranched polymers: from self-assembly to cytomimetic applications. Chem. Soc. Rev. 41, 5986–5997. 10.1039/c2cs35130g 22797315

[B6] Lee H. B. Bae C. W. Duy L. T. Sohn I. Y. Kim D. I. Song Y. J.. (2016). Mogul-patterned elastomeric substrate for stretchable electronics. Adv. Mater. 28, 3069–3077. 10.1002/adma.201505218 26917352

[B7] Li D. W. Niu Y. Yang Y. Wang X. Yang F. Shen H.. (2015). Synthesis and self-assembly behavior of POSS-embedded hyperbranched polymers. Chem. Commun. 51, 8296–8299. 10.1039/C5CC01338K 25876926

[B8] Mason T. O. Michaels T. C. Levin A. Gazit E. Dobson C. M. Buell A. K.. (2016). Synthesis of nonequilibrium supramolecular peptide polymers on a microfluidic platform. J. Am. Chem. Soc. 138, 9589–9596. 10.1021/jacs.6b04136 27387359

[B9] Newkome G. R. Moorefield C. N. (2015). From 1 → 3 dendritic designs to fractal supramacromolecular constructs: understanding the pathway to the Sierpinski gasket. Chem. Soc. Rev. 44, 3954–3967. 10.1039/C4CS00234B 25316287

[B10] Ni B. Huang M. Chen Z. Chen Y. Hsu C. H. Li Y.. (2015). Pathway toward large two-dimensional hexagonally patterned colloidal nanosheets in solution. J. Am. Chem. Soc. 137, 1392–1395. 10.1021/ja511694a 25590361

[B11] Palivan C. G. Goers R. Najer A. Zhang X. Car A. Meier W. (2016). Bioinspired polymer vesicles and membranes for biological and medical applications. Chem. Soc. Rev. 45, 377–411. 10.1039/C5CS00569H 26563574

[B12] Qian Q. Y. Xu J. Zhang M. Z. He J. L. Ni P. H. (2019). Versatile construction of single-tailed giant surfactants with hydrophobic poly(ε-caprolactone) tail and hydrophilic POSS head. Polymers 11:311. 10.3390/polym11020311 30960295 PMC6419185

[B13] Stefik M. Guldin S. Vignolini S. Wiesner U. Steiner U. (2015). Block copolymer self-assembly for nanophotonics. Chem. Soc. Rev. 44, 5076–5091. 10.1039/C4CS00517A 25856171

[B14] Tan K. W. Jung B. Werner J. G. Rhoades E. R. Thompson M. O. Wiesner U. (2015). Transient laser heating induced hierarchical porous structures from block copolymer–directed self-assembly. Science 349, 54–58. 10.1126/science.aab0492 26138971

[B15] Wang X. Gao P. Yang Y. Guo H. Wu D. (2018a). Dynamic and programmable morphology and size evolution via a living hierarchical self-assembly strategy. Nat. Commun. 9:2772. 10.1038/s41467-018-05142-3 30018381 PMC6050331

[B16] Wang X. Li D. Yang F. Shen H. Li Z. B. Wu D. C. (2013). Controlled cross-linking strategy: from hybrid hydrogels to nanoparticle macroscopic aggregates. Polym. Chem. 4, 4596–4600. 10.1039/c3py00811h

[B17] Wang X. Yang Y. Zuo Y. Yang F. Shen H. Wu D. (2016). Facile creation of FRET systems from a pH-responsive AIE fluorescent vesicle. Chem. Commun. 52, 5320–5323. 10.1039/C6CC01706A 27001923

[B18] Wang X. Yang Y. Y. Fan L. F. Yang F. Wu D. C. (2018b). POSS-embedded supramolecular hyperbranched polymers constructed from a 1 → 7 branching monomer with controllable morphology transitions. Sci. China Chem. 61, 311–318. 10.1007/s11426-017-9168-3

[B19] Wang X. Yang Y. Y. Gao P. Y. Yang F. Shen H. Guo H. X. (2015). Synthesis, self-assembly and photoresponsive behavior of tadpole-shaped azobenzene polymers. ACS Macro Lett. 4, 1321–1326. 10.1021/acsmacrolett.5b00698 35614776

[B20] Wu D. C. Loh X. J. Wu Y. L. Lay C. L. Liu Y. (2010). ‘Living' controlled *in situ* gelling systems: thiol-disulfide exchange method towards tailor-made biodegradable hydrogels. J. Am. Chem. Soc. 132, 15140–15143. 10.1021/ja106639c 20929223

[B21] Xia D. Wei P. Shi B. Huang F. (2016). A pillar [6] arene-based [2] pseudorotaxane in solution and in the solid state and its photo-responsive self-assembly behavior in solution. Chem. Commun. 52, 513–516. 10.1039/C5CC08038J 26530453

[B22] Yang Y. Wang X. Yang F. Wang L. Wu D. C. (2018). Highly elastic and ultratough hybrid ionic-covalent hydrogels with tunable structures and mechanics. Adv. Mater. 30:1707071. 10.1002/adma.201707071 29577453

[B23] Zhang J. Li X. Li Y. Wang H. Ma C. Wang Y. Z.. (2018). Cross-linked nanohybrid polymer electrolytes with POSS cross-linker for solid-state lithium ion batteries. Front. Chem. 6:186. 10.3389/fchem.2018.00186 29888223 PMC5981318

[B24] Zhang J. Yang F. Shen H. Wu D. C. (2012). Controlled formation of microgels/nanogels from a disulfide-linked core/shell hyperbranched polymer. ACS Macro Lett. 1, 1295–1299. 10.1021/mz300489n 35607159

[B25] Zhang W. A. Muller A. H. E. (2013). Architecture, self-assembly and properties of well-defined hybrid polymers based on polyhedral oligomeric silsequioxane (POSS). Prog. Polym. Sci. 38, 1121–1162. 10.1016/j.progpolymsci.2013.03.002

