# Supplementary material for: Fabrication of Customized Nanogel Carriers From a UV-Triggered Dynamic Self-Assembly Strategy
Source: Front Chem. 2019 Nov 8;7:769. doi: 10.3389/fchem.2019.00769 (PMC6857700; doi:10.3389/fchem.2019.00769)
Supplement: Supplementary file 1 [file Data_Sheet_1.PDF]

Fabrication of Customized Nanogel Carriers from a UV-Triggered Dynamic Self-Assembly Strategy

Wuren Bao[1], Jieran Lyu[2,5], Chunlin Li[3], Jifeng Zhang[4], Tunan Sun[3], Xing Wang[5,6,*], Jin Zhou[3,*] and Dawei Li[3,*]

[1]School of Nursing, Inner Mongolia University for Nationalities, Huolinhe Street West, Kerchin District, Tongliao, Inner Mongolia, 028000, P.R. China

[2]Clinical Medicine Academy of Shandong First Medical University, Taian 271016, P.R. China

[3]The 8th Medical Center of Chinese PLA General Hospital, Beijing 100091, P.R. China

[4]Department of Orthopedic Surgery, Tongliao City Hospital, Kerchin Street, Kerchin District, Tongliao, Inner Mongolia 028000, P.R. China

[5]Beijing National Laboratory for Molecular Sciences, State Key Laboratory of Polymer Physics & Chemistry, Institute of Chemistry, Chinese Academy of Sciences, Beijing 100190, P.R. China

[6]University of Chinese Academy of Sciences, Beijing 100049, P.R. China

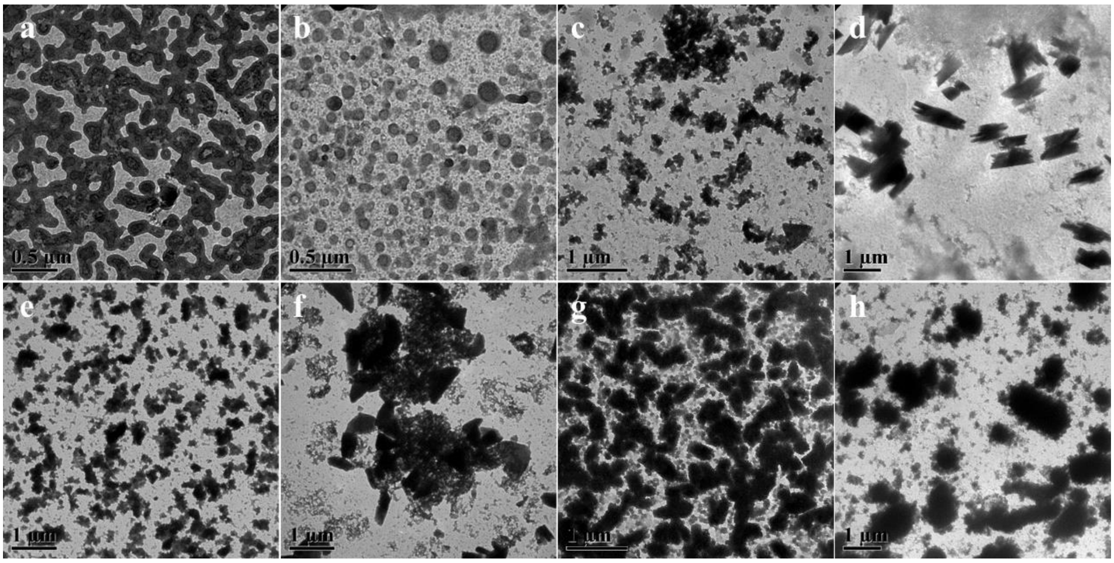

Fig. S1 TEM images showing morphology and size evolution with a concentration of 1 mg/mL aqueous media under UV irradiation at (a) 2, (b) 6, (c) 10, (d) 12, (e) 16, (f) 28, (g) 36 and (h) 44 h.
